# Brain Asymmetry Detection and Machine Learning Classification for Diagnosis of Early Dementia †

**DOI:** 10.3390/s21030778

**Published:** 2021-01-24

**Authors:** Nitsa J. Herzog, George D. Magoulas

**Affiliations:** 1 Department of Computer Science, Birkbeck College, University of London, London WC1E 7HZ, UK; nitsa@dcs.bbk.ac.uk; 2 Birkbeck Knowledge Lab, University of London, London WC1E 7HZ, UK

**Keywords:** asymmetry detection, brain asymmetry, brain MRI, dementia, machine learning methods, SVM, deep learning

## Abstract

Early identification of degenerative processes in the human brain is considered essential for providing proper care and treatment. This may involve detecting structural and functional cerebral changes such as changes in the degree of asymmetry between the left and right hemispheres. Changes can be detected by computational algorithms and used for the early diagnosis of dementia and its stages (amnestic early mild cognitive impairment (EMCI), Alzheimer’s Disease (AD)), and can help to monitor the progress of the disease. In this vein, the paper proposes a data processing pipeline that can be implemented on commodity hardware. It uses features of brain asymmetries, extracted from MRI of the Alzheimer’s Disease Neuroimaging Initiative (ADNI) database, for the analysis of structural changes, and machine learning classification of the pathology. The experiments provide promising results, distinguishing between subjects with normal cognition (NC) and patients with early or progressive dementia. Supervised machine learning algorithms and convolutional neural networks tested are reaching an accuracy of 92.5% and 75.0% for NC vs. EMCI, and 93.0% and 90.5% for NC vs. AD, respectively. The proposed pipeline offers a promising low-cost alternative for the classification of dementia and can be potentially useful to other brain degenerative disorders that are accompanied by changes in the brain asymmetries.

## 1. Introduction

Dementia is a brain disorder that affects normal brain function due to the loss of neurons or neurons’ functionality. Dementia may include a group of symptoms such as memory loss, lack of reasoning and judgment, problems with speech and understanding language, and changes in personality [1]. A total of 46.8 million people worldwide have dementia, and approximately 9.9 million new cases are registered every year. The proportion of dementia in the general population aged 60 and over is 7.1%.

There are several types of degenerative diseases accompanied by dementia. Alzheimer’s disease (AD) is the most common one, followed by vascular dementia, Lewy body dementia, Frontotemporal dementia, Parkinson’s disease, and Huntington’s disease. 

Early dementia or amnestic Mild Cognitive Impairment (aMCI) is characterized by minor problems with memory, speech, or decision-making. More than 80% of people satisfying the definition of aMCI progress to Alzheimer’s disease within 6 years. Early detection and identification of the structural and functional cerebral changes are crucial for providing proper care and treatment. Computer-aided diagnosis (CAD) of the anatomical changes in the brain, based on magnetic resonance imaging (MRI), gives accurate results for early diagnosis of brain disorders [2,3].

The current study is based on the hypothesis that brain asymmetry changes as a result of the development of early and progressive dementia. The evaluation of asymmetries in the cortex of the brain is based on structural Magnetic Resonance Imaging (sMRI). This research aims to investigate the pattern of these changes using MRI and computer vision techniques. The paper proposes an algorithm for segmenting and visualizing the differences in the symmetry between right and left hemispheres of the brain and generating asymmetry features. This is mainly focused on the early changes in the brain cortex when the clinical diagnosis is not obvious and cannot be done by medical professionals using traditional diagnostic methods. The pattern of brain asymmetries in a group of patients with normal cognition (NC), mild cognitive impairment (MCI) and dementia, very mild stable, and progressive to Alzheimer’s Disease (AD) is analyzed with the help of statistical features. The information collected from image asymmetries can be sent for further processing and classification. The paper verifies the robustness of the generated brain asymmetry images and features, demonstrating their potential to produce consistent performance across different classification models. The rest of the article is organized as follows. Section 2 describes the problem and provides a review of the area. Section 3 introduces the Alzheimer’s Disease Neuroimaging Initiative (ADNI) database used in this study and derives the data processing pipeline, including the approach for detection and analysis of image asymmetries and the machine learning methods used for classification. Results are presented in Section 4. The paper ends with a general discussion of the area and directions for future research in the field of brain asymmetries.

## 2. Background of the Study

The investigation of the anatomical properties and functional ability of the human brain is an intensively developing research area. The human brain has an overall leftward posterior and rightward anterior asymmetry (known as Petalia and Yakovlevian torque) [4]. The right cerebral hemisphere protrudes forward, and the left hemisphere protrudes backward compared to the right one. This type of asymmetry is mostly found in right-handed individuals—around 90% of the human population is right-handed [5]—while the opposite pattern is observed in left-handed individuals. The brain asymmetry is associated with lateralization that is a structural and functional difference between the left and right sides of the brain. These asymmetries originate from genetic and epigenetic factors in the evolutionary development of the brain [6,7]. The brain structure is more lateralized in males than in females [8]. It also depends on the age of the person. The brain activity in the frontal lobes of young adults is more lateralized than in elderly healthy individuals, whose brain becomes more symmetrical with binarized activities in both hemispheres [9,10]. The degree of structural asymmetry is correlated with the degree of functional lateralization. The left hemisphere is mostly responsible for language processing and logical thinking. For example, it includes the Broca’s speech area and the Wernicke’s language comprehension area. The right hemisphere specializes in musical and artistic abilities, spatial recognition, attention, and emotions [7].

The analysis of brain symmetry helps in the diagnosis of brain-related disorders [11]. The brain regions show a progressive decrease in the degree of asymmetry in patients with Mild Cognitive Impairment (MCI) and an increase in asymmetry in patients with Alzheimer’s disease (AD) [12]. Left-hemisphere regions are affected earlier and more severely. The right cerebral regions become dominant in AD patients, but not in the early phase of the MCI. The abnormal hemispheric asymmetries of AD and MCI patients significantly correlate with functional brain activities and memory performance. Patients with MCI show an increase in the activation of many brain regions in the right hemisphere during the processing of word memory tasks [13]. Those areas are compensatorily activated compared to the activation zones in the left hemisphere of the healthy controls. The degree of asymmetry is not the same in the different parts of the brain [14]. Progressive dementia in patients with Alzheimer’s disease is associated with a significant increase in the neuroanatomical asymmetries in subcortical brain structures such as the hippocampus and amygdala [15,16]. The research findings prove that shape analysis can detect the progression of dementia earlier than volumetric measures. Shape asymmetry, based on longitudinal asymmetry measures in the hippocampus, amygdala, caudate, and cortex can be a powerful imaging biomarker for the early presymptomatic prediction of dementia.

As with other types of imaging data processing, segmentation techniques are a necessity for detailed study of the anatomical regions of the brain and their symmetries. Brain segmentation is an important task in brain analysis and often the most critical step in many medical applications. Nowadays, many segmentation techniques offer the detection and separation of the whole brain tissues from the skull (skull stripping) or partial separation of the specific regions (gray or white matter) and individual brain structures in accordance with the anatomical atlas [17]. The current study proposes an algorithm for segmentation of the hemispheric asymmetries. The segmented areas usually require additional analytical tools for understanding their property in depth. Feature engineering gives knowledge about the most remarkable characteristics of the image [18]. Features detection and description refers to the procedure of identifying points of interest in an image (or object) that can be used to describe and analyze the image (object) contents [19], providing valuable data for image analysis. The current project includes an analysis part that is based on the evaluation of the statistical properties of imaging data. Statistical description of the image texture can generate a number of relevant and distinguishable features, which is crucial for the interpretation of the research findings. 

Several studies in the machine learning area have highlighted the importance of feature selection for the improvement of classification performance [20,21]. For example, an effective feature selection method based on computing the chi-square statistical value was introduced in [22]. The diagnostic system of heart disease based on feature fusion, feature selection, and weighting techniques gives a high prediction of 98.5% [23]. Zhou et al. [24] applied the C4.5 statistical (decision tree) classifier to select those weights from the gray matter (GM) regions that were most affected by the atrophic process. Using feature selection, the researchers were able to improve the classification performance of the proposed Transfer AdaBoost algorithm and achieved a classification accuracy of 85.4% for the ADNI database and 93.7% for a local hospital dataset in the diagnosis of AD and MCI. *t*-test score ranking of the extracted features was proposed by Beheshti et al. [21] in a research work about discriminating between stable and progressive MCI cases. A genetic algorithm, equipped with the Fisher criterion function [25], evaluated the separation between the two groups of data and helped to select the most discriminative feature subsets for the classification with linear Support Vector Machine (SVM). The feature selection process raised the accuracy of the classification by 16%. The average calculated accuracy reached 93.01% for stable MCI and 75% for progressive MCI. Another feature engineering technique combined with a Regularized Extreme Learning Machine (RELM) algorithm was proposed by Lama et al. [26]. The high-level PCA features [27] were chosen using the softmax function (a function that takes a vector of real numbers as input and normalizes it into a probability distribution). The proposed method showed 75.33% of accuracy for AD and 80.32% for MCI with binary classification and 76.61% with multiclass classification. Glozman and Le [28] developed machine learning methods, based on the architecture of white matter tracts, to classify Alzheimer’s and healthy subjects. The ranking process was based on the differences in the average population values for each feature, whilst feature dimensionality was reduced using PCA. The researchers reported an increase in performance up to 92% after applying feature normalization and ranking. 

Another popular method for the improvement of classification performance is the implementation of classifier ensembles [29]. They combine multiple meta-algorithms in one predictive model in order to minimize error and enhance predictive accuracy. Moradi et al. [30] combined features collected from imaging data (MRI biomarkers) with age and neuropsychological test results with a Random Forest (RF) classifier [31]. Aggregated biomarkers raised the classification accuracy by 5.5% (from 76.5% to 82%). Grassi et al. [32] investigated sociodemographic information, clinical characteristics, neuropsychological measures of 550 subjects and combined the results using supervised ensemble learning. Each parameter from the multiple data was tested with 13 machine learning techniques, including hyperparameters optimization and cross-validation procedures. All the initially selected categorical features (14 continuous, 2 dichotomous, and 1 polytomous) were weighted, ranked, and organized into site-independent, stratified sub-sets. The most discriminative features were selected and contributed to the final result, which showed an AUROC (Area Under the Receiver Operating Characteristics) of 0.88, a sensitivity of 77.7%, and a specificity of 79.9%.

Deep learning methods have received wide popularity in many domains including image processing and analysis. Their popularity is based on the fact that, in most cases, they did not require image preprocessing and feature engineering prior to the classification process. These algorithms work well with large and biased data. A convolutional neural network is a class of deep neural networks that was specially designed to work with imaging data. It can distinguish between AD and NC with an accuracy of 98% and predict a stable or converted MCI with an accuracy of 75% while processing and training only MRI data [33]. Other neural network models were proposed for the processing of non-imaging medical data. For example, Stamate et al. [34] created three deep learning models for processing and analysis of clinical and genetic data, and several parameters collected from MRI and PET images. Their models achieved 86% accuracy in the classification of dementia and cognitive impairment. 

Table 1 summarizes state-of-the-art methods and their results for the diagnosis of Mild Cognitive Impairment and Alzheimer’s Disease using the ADNI database.

In contrast with previous work (see Table 1), this study proposes a new approach to the early diagnosis of dementia. The pipeline includes an asymmetry segmentation algorithm that visualizes the differences between the right and the left hemispheres of the MRI slices of the brain. The features are collected directly from the regions that have been already affected by the degenerative atrophic processes. This approach simplifies the feature engineering stage and gives an advantage over other state-of-the-art methods mentioned above, where feature collection and selection processes are more complicated and time-consuming. The images of asymmetries take less memory space than original MRIs. For example, two images (original and asymmetry) in the same file type and dimensions have sizes of 55.6 KB and 18.7 KB, respectively. This reduces the storage of the imaging data and speeds up the classification processing of large datasets that make use of images as an input. The proposed method, unlike the methods above, does not require special hardware equipment and cloud computations. Experiments, presented in detail in Section 4, provide evidence that it performs well even without fine-tuning the classification models. For example, the prediction of early mild cognitive impairment compared to the normal controls using SVM is 92.5%, which appears quite promising compared with the accuracy of other fine-tuned models that is in the range between 80.32% and 92%, as shown in Table 1. 

## 3. Materials and Methods

### 3.1. Data Repositories and Participants

Data used in the preparation of this article were obtained from the Alzheimer’s Disease Neuroimaging Initiative (ADNI) database (adni.loni.usc.edu), which was launched in 2003 as a public-private partnership led by Michael W. Weiner, MD. The primary goal of ADNI has been to test whether serial magnetic resonance imaging (MRI), positron emission tomography (PET), other biological markers, and clinical and neuropsychological assessment can be combined to measure the progression of mild cognitive impairment (MCI) and early Alzheimer’s disease (AD); for up-to-date information, see www.adni-info.org (Supplementary Materials).

The datasets of T1-waited images have been created using MRI data of 750 subjects aged between 55 and 75 years. Patients are divided equally into groups of subjects with normal cognition (NC), early mild cognitive impairment (EMCI), and Alzheimer’s Disease (AD). 

### 3.2. Research Methods

The data processing pipeline, including image processing and machine learning classification, has been implemented in Matlab using affordable and easy-to-obtain commodity hardware. The supported software and hardware characteristics are as follows: Windows 10 Enterprise, processor—Intel (R) Core (TM), i7-7700 CPU@ 3.60 GHz, 16 GB RAM.

The methods for early diagnosis of dementia include detection of image asymmetries, statistical feature extraction, and image analysis, machine learning algorithms.

**Figure 1:**
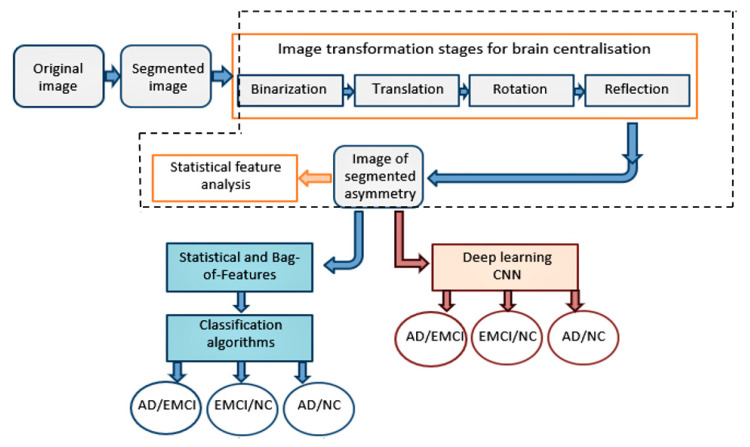
The MRI data processing pipeline includes image transformation stages with the following feature extraction and machine learning classifications.

Figure 1 illustrates the stages of data processing, analysis, and classification of the pipeline, whilst the dashed line accentuates the contribution of this paper.

#### 3.2.1. Image Preprocessing

All images in the dataset were normalized and resized to 256-by-256-by-3 pixels. Then, brain tissues were segmented manually from the skull using adjusted upper and lower boundaries of a threshold level of the pixel values. This simple algorithm can be replaced with higher0quality brain segmentation software [35,36].

#### 3.2.2. Detection of Image Asymmetry

A key stage of the pipeline is the detection of the vertical line of symmetry in the brain. As soon as that line is found, it is possible to flip the brain image from the left to the right across the vertical axis and extract the image asymmetries. A flipped or reverse image is an image that is mirrored across the horizontal or vertical axis [37].

The hypothesis being tested in this part of the research is that there is an axis of reflective symmetry running through the center of the brain [38]. Thus, it is necessary to find the center of the brain and translate it to the center of the image. At the same time, the brain needs to be rotated to the correct angle to reach the maximum symmetry in the image. The algorithm was tested on single slices of the brain. The same idea can be extended and applied to the whole 3D brain.

The brain center is allocated using an image binarization technique and calculating the image centroid [39]. Image binarization [40] is the process of converting a grayscale image to black and white. As a result, 256 shades of the grayscale image are reduced to 2 colors only. The binarization is done according to the level of a threshold. All pixels in the image above the threshold level are replaced by the value 1 (white) and other pixels that are below that level by the value 0 (black). 

In the context of image processing and computer vision, the centroid is the weighted average of all the pixels in an image. The “weighted” centroid, or center of mass, is always at the exact center and depends on the gray levels in the image.

**Figure 2:**
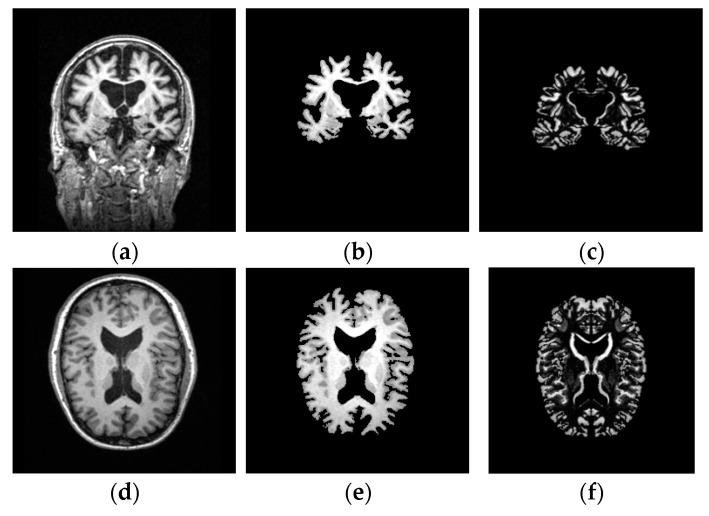
T1-waited brain MRI processing in 2 axes of symmetries: (**a**) Original image in the coronal plane; (**b**) Segmented image of the brain tissues in the coronal plane; (**c**) Image asymmetry in the coronal plane; (**d**) Original image in the axial plane; (**e**) Segmented image of the brain tissues in the axial plane; (**f**) Image asymmetry in the axial plane.

Figure 2 illustrates the main stages of the image processing prior to classification.

**Figure 3:**
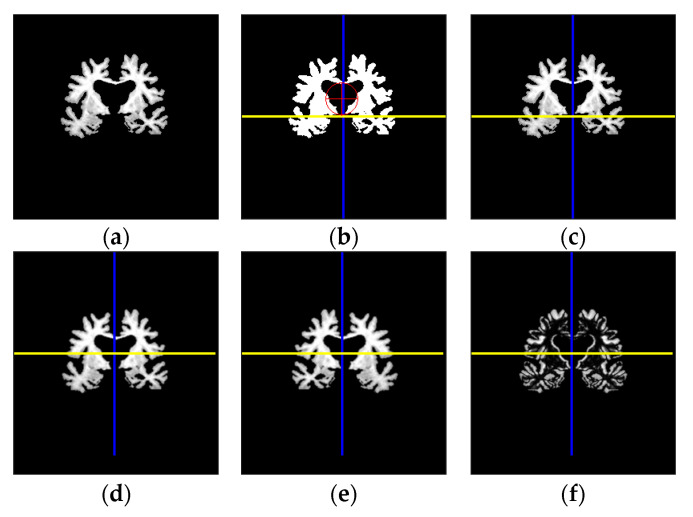
Image transformation stages: (**a**) Segmented grayscale image; (**b**) Centers of the binary image; (**c**) Image translated to the middle of the vertical axis; (**d**) Centered and rotated image with respect to 2 axes of symmetry; (**e**) Reflected image via vertical axis; (**f**) Image asymmetry.

Detailed image transformation stages are provided in Figure 3.

The last image is obtained as a result of the mirroring of the left-brain hemisphere to the right and right-brain hemisphere to the left, which is followed by subtraction of the hemispheres from each other. This process can be expressed symbolically as an equation:

D = (L − R) + (R − L),(1)

where D is an image asymmetry, L is an image matrix of the left hemisphere, R is an image matrix of the right hemisphere.

**Figure 4:**
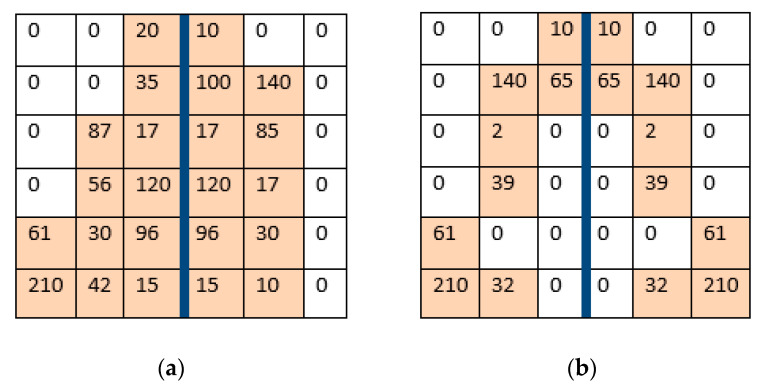
Matrices of gray-scale image: (**a**) Initial matrix; (**b**) Matrix of segmented asymmetry mirrored via the vertical axis.

Figure 4 illustrates the pixel transformation values from segmentation of asymmetry in a small image of size 6-by-6. The numbers in the cells correspond to the gray level of the pixel values.

The symmetrical image areas (Figure 4b) become equal to 0 due to matrix subtraction. They are visualized as black areas in the image. The asymmetrical parts of the image are represented as different intensity gray levels from 1 to 255.

#### 3.2.3. Generating Asymmetry Features 

The statistical feature approach for representing image properties is well-known in image processing [41]. Extracted statistical features represent the color, texture, or morphological properties of an image. Ten strong and stable statistical features, namely MSE (Mean Squared Error), Mean, Std (Standard deviation), Entropy, RMS (Root Mean Square), Variance, Smoothness, Kurtosis, Skewness, and IDM (Inverse difference moment) [42,43,44,45,46,47,48], are chosen for the research and combined in vectors representing the MRI data properties. These features give information about the likelihood of gray pixel values in a random position in an image, their orientation, and interaction with other surrounding pixels [24]. In our pipeline, images with segmented asymmetry have been analyzed to generate statistical features from the image differences using discrete wavelet transform (DWT) [49,50]. The only exception is the first feature on the list, Mean Squared Error, which has been calculated as a difference between the original image and its mirrored version without wavelet transformation.

As an example, the features vector, extracted from the image asymmetry of a patient’s MRI slice, is provided below:

[849.477703   10.47024065   90.10031233   0.764676239   51.73779963   7260.005288   0.999995013   52.78224277   5.661092548   9052.865381]

**Figure 5:**
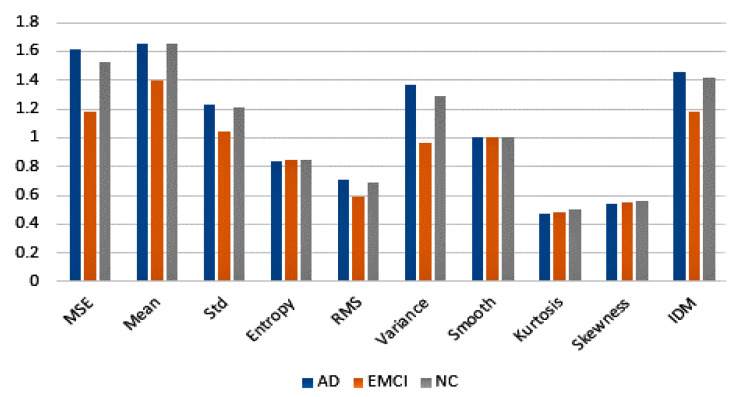
Statistical mean of each image asymmetry feature for normal cognition (NC), early minor cognitive impairment (EMCI), and Alzheimer’s disease (AD) patients.

Figure 5 shows features averaging based on normalized (from 0 to 1) features data extracted from a set of 300 images with segmented asymmetry, which were generated from a set of 300 MRI slices from different patients equally distributed in each group.

**Figure 6:**
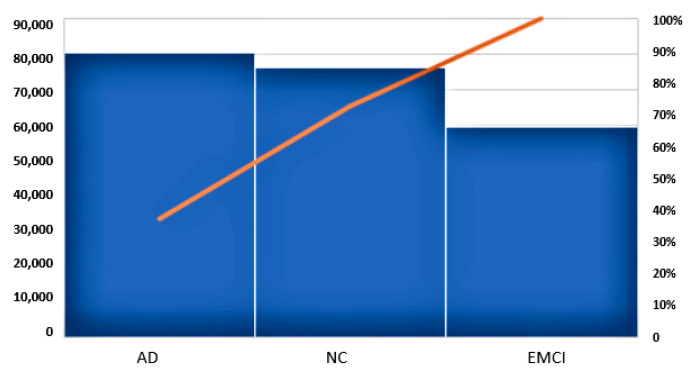
Pareto chart of MSE for the three classes. The primary axis, on the left, shows the total MSE feature value, whilst the secondary axis, on the right, shows the cumulative percentage of the total value for each class.

Figure 6 provides an MSE feature analysis with a Pareto chart. The MSE value for each class has been calculated from the differences between the original image and its mirrored version for all images in that class and indicates the impact of asymmetry for each class. The highest MSE bar for AD confirms that changes in symmetry in the MRI slices of this image group are substantial compared to changes in symmetry in the MRI slices of the other groups, with the EMCI group’s images looking more “symmetrical” than the others. The cumulative line on the secondary axis shows the contribution of each bar (image class) in the total value as a percentage.

Further analysis of image asymmetries based on 10 statistical features (Figure 5) confirms that the above observations are present in other datasets and can be extended to different sizes of data (MRI slices). These findings support the view that image asymmetry decreases in the initial stage of the generative process in the brain (Early Mild Cognitive Impairment) and grows when the person develops moderate and severe dementia (Alzheimer’s disease).

Statistical features collected from image asymmetries were enriched with Bag-of-Features (BOF) to get the most detailed image “signatures”. State-of-the-art literature confirms the high performance of the Bag-of-Features algorithm in image classification [51]. BOF [52] based on the Speeded Up Robust Features (SURF) [53,54] and the K-Means clustering [55] algorithms were represented as vectors of occurrence of the local image features in this work. 

#### 3.2.4. Classification Using Machine Learning 

In this stage of the pipeline, various machine learning methods were trialed as reported in the next section. The purpose was to verify the robustness of brain asymmetry image and asymmetry features for early diagnosis of dementia when applied to different classification models without fine-tuning their hyperparameters. Supervised machine learning was the main focus, and the following binary classifiers were used: Naïve Bayes (NB) [56], Linear Discriminant (LD) [57], Support Vector Machine (SVM) (linear, quadratic, cubic, Medium Gaussian kernels) [58,59], K-Nearest Neighbor (fine, cosine kernels) [60]. All models from the list were trained using Matlab Classification Learner App, except the NB classifier, which was trained separately. Table 2 presents the models default hyperparameter values used in the experiments.

The potential of transfer learning was also explored by applying a type of Convolutional Neural Network (CNN), the so-called AlexNet [61,62]. This is a convolutional neural network with 8 deep layers. The network has five convolutional layers and three fully connected layers. The 1-st layer requires input image of size 227-by-227-by-3, where 3 is the number of color channels.

**Figure 7:**
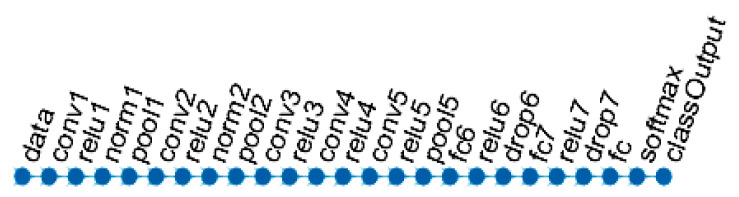
Adapted AlexNet architecture used in the experiments.

Figure 7 displays the network architecture. The last 3 layers of AlexNet were preliminary configured to 1000 classes as it was trained to solve a different classification problem. To adopt the network for the current classification task, the last 3 layers of the AlexNet were replaced with a fully connected layer, a softmax layer, and a binary classification output layer. 

## 4. Experiments and Results

Six hundred images of brain asymmetries with equal number of AD, EMCI, and NC subjects were combined into three binary datasets, EMCI vs. NC, AD vs. NC, and AD vs. EMCI. The datasets include images in 2 dimensions (planes): vertical or frontal and horizontal or axial. The purpose of the experiment was to investigate whether asymmetry features produce consistent performance across different classification models, and no fine-tuning or model optimization was performed.

The performance of NB, LD, SVM, and KNN trained models was estimated using 10 simulation runs of a 10-fold cross-validation procedure, while for the adapted AlexNet (CNN), 2 simulation runs were conducted without using cloud infrastructure. Training and evaluation of the CNN are also processed differently from the rest of methods: images of segmented asymmetry were resized to 227 × 227 × 3 and fed into the model with 80% of the images used for training, 10% for validation, and 10% for testing. The parameters of the CNN were 128 mini-batch size, 10 epochs, validation data frequency of 50. Each CNN training/validation/testing round took approximately 43 min on our hardware, while SVMs were trained for 10 epochs, with each epoch taking approximately 5 s. All the machine learning models were tested on unseen data, and Table 3 summarises the average performance (%) in testing of the early mild cognitive impairment, normal cognition, and Alzheimer’s disease datasets of the ADNI database. The highest results for each dataset are shown in bold.

The test results show that features extracted from asymmetries provide consistent performance across different classification models without model-specific fine-tuning of hyperparameters. The SVM variants and the LD method can become the methods of choice as they can be easily trained on commodity hardware and demonstrate better accuracy than other alternatives in all cases. The best performance amongst the SVM variants was shown by SVMs with the polynomial cubic and quadratic kernel (C-SVM and Q-SVM). C-SVM accuracy of EMCI vs. NC was 92.5%, sensitivity was 95.0%, specificity was 90.0%; accuracy of AD vs. NC was 93.0%, sensitivity was 93.0%, specificity was 93.0%; accuracy of AD vs. EMCI was 86.5%, sensitivity was 88.0%, specificity was 85.0%. Q-SVM accuracy of EMCI vs. NC was 92.5%, sensitivity was 92.0%, specificity was 93.0%; accuracy of AD vs. NC was 92.5%, sensitivity was 90.0%, specificity was 95.0%; accuracy of AD vs. EMCI was 86.5%, sensitivity was 89.0%, specificity was 84.0%. The prediction results of the CNN are in the same range as those of the other classifiers.

An aggregated measure, the area under the ROC Curve (AUC), shows the relationship between data sensitivity and specificity across different levels of threshold [63], giving an additional view on the classifier performance. AUC results of the best available models from C-SVM and CNN are presented in Table 4. 

**Figure 8:**
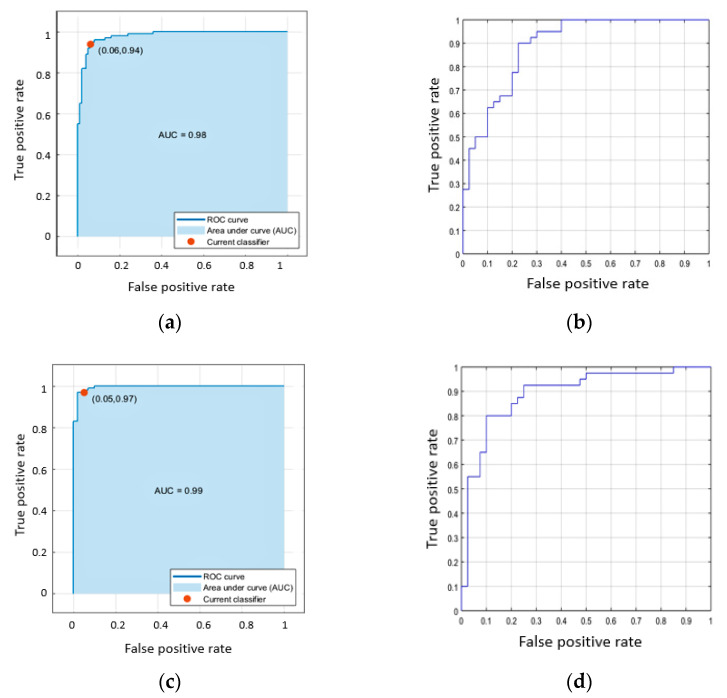

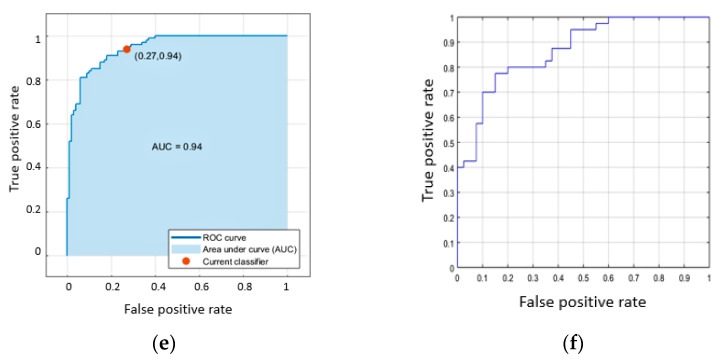
AUC/ROC curves of Cubic Support Vector Machine (C-SVM) (left) and Convolutional Neural Network (CNN) (right) classifiers: (**a**,**b**) is EMCI vs. NC; (**c**,**d**) is AD vs. NC; (**e**,**f**) is AD vs. EMCI.

Figure 8 presents the AUC/ROC curves of three binary datasets for the C-SVM and CNN classifiers. All the sub-figures were created on Matlab during data testing.

In general, satisfactory performance was obtained in classification between EMCI and NC, AD and NC, and AD and EMCI. In this context, it is worth noting an important difference between the CNN classifier and all other methods: the CNN is the only model that operates directly on images of segmented asymmetry, whilst all other models operate on images with segmented asymmetry that have been analyzed to generate statistical features using discrete wavelet transform (DWT). It is expected that fine-tuning and model optimization can potentially improve the performance of all classification models further. This will be the subject of another communication of ours, since the focus of the current study has been on demonstrating the potential of brain asymmetry images and features. In the next section, the classification performance using asymmetry features is discussed further in the context of the literature on the diagnosis of dementia.

## 5. Discussion and Conclusions

The paper introduced a new diagnostic approach based on analysis of brain asymmetry for early classification of initial dementia when clinical symptoms are very mild and challenging. The early changes in the brain asymmetries in subjects with early mild cognitive impairment are accompanied by an increase in the symmetry between the left and the right hemisphere. The proposed pipeline is based on the detection of the image asymmetries using magnetic resonance imaging. The study involved the imaging resources of the well-known ADNI medical imaging database. The experimental study presented in the previous section included cross-sectional classification of early mild cognitive impairment, Alzheimer’s disease, and cognitively normal subjects from the ADNI database. The experimental results support the hypothesis that changes in the brain asymmetries during the development of dementia convey important information (see [5]), as they were used to generate useful features for classification. 

In contrast to other methods in the literature (see Section 2), this study is less complex in term of image processing. For example, the image processing time on average was 0.1 min per image (3.6 GHz Intel Core i7, 16 GB RAM), compared to the article [30], where it took 8 min (3.4 GHz Intel Core i7, 8 GB RAM). The images of segmented asymmetries require 3 times less memory space than similar originals. This potentially gives an advantage in terms of computational time spent on training a classifier, although it is not possible to make a direct comparison with other approaches in the literature as hardware specifications differ. Nevertheless, it is worth mentioning that the CPU time for an asymmetry features-trained single classifier, including 10-fold cross-validation, was approximately 5 s on our hardware, which appears considerably low compared to other methods that used more complex feature sets [31]. Classification performance achieved in this paper is comparable with the results obtained by other researchers, summarized in Table 1 for the ADNI database. In particular, predictive accuracy of 92.5% for EMCI vs. CN and 93% for AD vs. CN is high, especially when considering the complexity of the schemes shown in this table (e.g., a larger number of features, a higher number of architectural parameters, and so on). Further performance improvement can be potentially achieved with SVM and CNN classifiers by optimization of architectures, as demonstrated in other works in the literature [29,32]; a study of this type is included in our immediate plans.

A limitation of this study is that it did not investigate handedness and its influence on the classification results, as the images downloaded from the ADNI database were not separated according to the handedness. Thus, a potential direction for further work concerns an investigation of the role and the impact of asymmetric differences between the right- and left-handed patients. In addition, the potential of deep learning models deserves further study. Lastly, a longitudinal study inside the group of patients with mild cognitive impairment could help to distinguish between stable and progressive dementia and monitor further changes in the brain due to the development of the disease. This asymmetrical changes in the brain can be tested with other psychiatric conditions such as schizophrenia and Parkinson’s Disease.

On a practical level, the use of image asymmetry and asymmetry features has potential to contribute to the design of end-user (e.g., physicians and general medical practitioners) applications, which will run on commodity hardware, for early diagnosis of cognitive decline and investigation of the nature of the structural changes in the brain. These applications can exploit the generalization properties of machine learning models on unseen MRI data, as demonstrated in this study.

## Figures and Tables

**Table 1:** State-of-the-art methods of diagnosis of Mild Cognitive Impairment and Alzheimer’s Disease.

Authors	Methods	Results
Lama et al. [26]	PCA Features + Regularized Extreme Learning Machine (unsupervised classification learning algorithm based on single hidden-layer feedforward neural networks) of MRI (AD, MCI, NC).	Accuracy: 80.32% (for binary classification), 76.61% (for multiclass.)
Zhou et al. [20]	Transfer Learning Method (includes Transfer AdaBoost algorithm) + C4.5 classifier of MRI (AD, MCI, NC.)	Accuracy: 85.4% (improves with optimized feature selection).
Beheshti et al. [21]	Feature-ranking + genetic algorithm + SVM classifier of MRI (AD, MCI).	Accuracy: 93.01% (stable MCI), 75% (progressive MCI), 78.94% (without feature selection), 94.73% (with feature selection).
Moradi et al. [30]	Logic regression + MRI biomarker (based on low-density separation) + SVM + neuropsychological test results + random forest classifier of MRI (AD, MCI, NC).	MRI + cognitive test improves the accuracy by 5.5% (from 76.5% to 82%).
Glozman and Le [28]	Feature ranking of the white matter (WM) + SVM (with Linear and RBF Kernels) and Logic Regression of DTI (AD).	Average accuracy: 92%.
Grassi et al. [32]	Ensemble algorithm using sociodemographic information, clinical characteristics, neuropsychological measures; supervised ML. (Conversion from MCI to AD).	AUROC: 0.88; sensitivity: 77.7%; specificity: 79.9%. Range of AUROC for proposed models is 0.83–0.90.
Basaia et al. [33]	CNNs; classification of AD, stable MCI and converted MCI. Did not use feature engineering.	Accuracy of AD vs. CN: 98%; sMCI vs. cMCI: 75%.
Stamate et al. [34]	Deep Learning models: two Multi-Layer Perceptron (MLP1 and MLP2) models and a Convolutional Bidirectional Long Short-Term Memory (ConvBLSTM) model. The features were collected from clinical and genetic data, MRI data, PET data and some additional biospecimen. (Dem, MCI, CN).	The best models (MLP1 and MLP2) show the accuracy 0.86 for Dem, MCI, and CN classes.

**Table 2:** Hyperparameters of the machine learning algorithms.

Model	Hyperparameters
NB	Distribution: normal (Gaussian)
LD	Discriminant type: linear
L-SVM	Kernel function: linear Box constraint level:1 Kernel scale mode: auto Standardize data: true
Q-SVM	Kernel function: quadratic Box constraint level:1 Kernel scale mode: auto Standardize data: true
C-SVM	Kernel function: cubic Box constraint level:1 Kernel scale mode: auto Standardize data: true
MG-SVM	Kernel function: medium Gaussian Box constraint level:1 Kernel scale mode: manual Kernel scale: 32 Standardize data: true
Fine-KNN	Number of neighbors: 1 Distance metric: Euclidian Distance weight: equal Standardize data: true
Cos-KNN	Number of neighbors: 10 Distance metric: cosine Distance weight: equal Standardize data: true

**Table 3:** Average performance (%) of binary classifiers.

Datasets	NB	LD	L-SVM	Q-SVM	C-SVM	MG-SVM	Fine-KNN	Cos-KNN	CNN
EMCI vs. NC									
Accuracy	77.0	91.0	89.0	**92.5**	**92.5**	88.0	83.0	92.0	75.0
Sensitivity	78.0	91.0	89.0	92.0	95.0	85.0	99.0	96.0	90.0
Specificity	76.0	91.0	89.0	93.0	90.0	91.0	67.0	88.0	60.0
AD vs. NC									
Accuracy	78.5	90.0	92.0	92.5	**93.0**	90.0	86.5	89.5	90.0
Sensitivity	78.0	88.0	91.0	90.0	93.0	85.0	98.0	90.0	89.0
Specificity	79.0	92.0	93.0	95.0	93.0	95.0	75.0	89.0	92.0
AD vs. EMCI									
Accuracy	78.5	83.0	80.5	**86.5**	**86.5**	80.5	79.0	80.0	81.25
Sensitivity	75.0	85.0	84.0	89.0	88.0	84.0	78.0	83.0	72.5
Specificity	81.0	81.0	77.0	84.0	85.0	77.0	80.0	78.0	90.0

**Table 4:** AUC for cubic-SVM and CNN.

Datasets	C-SVM	CNN
EMCI vs. NC	0.98	0.90
AD vs. NC	0.99	0.92
AD vs. EMCI	0.94	0.88

## Data Availability

The MRI images used in the preparation of this article were obtained from the ADNI database (adni.loni.usc.edu), which is available for download from the Laboratory of Neuroimaging (LONI). Data use is subject to an Alzheimer’s Disease Neuroimaging Initiative (ADNI) data use agreement.

## Supplementary Materials

The MRI images are available online at www.adni-info.org. The source code is available at https://uk.mathworks.com/matlabcentral/fileexchange/85628-asymmetry-detection-of-the-mri-brain.
